# Plasmonic Optical Trapping in Biologically Relevant Media

**DOI:** 10.1371/journal.pone.0093929

**Published:** 2014-04-07

**Authors:** Brian J. Roxworthy, Michael T. Johnston, Felipe T. Lee-Montiel, Randy H. Ewoldt, Princess I. Imoukhuede, Kimani C. Toussaint

**Affiliations:** 1 Department of Electrical and Computer Engineering, University of Illinois at Urbana-Champaign, Urbana, Illinois, United States of America; 2 Department of Mechanical Science and Engineering, University of Illinois at Urbana-Champaign, Urbana, Illinois, United States of America; 3 Department of Bioengineering, University of Illinois at Urbana-Champaign, Urbana, Illinois, United States of America; University of Milano-Bicocca, Italy

## Abstract

We present plasmonic optical trapping of micron-sized particles in biologically relevant buffer media with varying ionic strength. The media consist of 3 cell-growth solutions and 2 buffers and are specifically chosen due to their widespread use and applicability to breast-cancer and angiogenesis studies. High-precision rheological measurements on the buffer media reveal that, in all cases excluding the 8.0 pH Stain medium, the fluids exhibit Newtonian behavior, thereby enabling straightforward measurements of optical trap stiffness from power-spectral particle displacement data. Using stiffness as a trapping performance metric, we find that for all media under consideration the plasmonic nanotweezers generate optical forces 3–4x a conventional optical trap. Further, plasmonic trap stiffness values are comparable to those of an identical water-only system, indicating that the performance of a plasmonic nanotweezer is not degraded by the biological media. These results pave the way for future biological applications utilizing plasmonic optical traps.

## Introduction

Optical tweezers, introduced by Ashkin in 1986 [1], have become an indispensable component in the biophysicists' toolkit, leading to breakthroughs in understanding DNA structure [2], RNA transcription [3], protein folding [4], cell motility [5], [6], and single-molecule biophysics [7], [8]. However, investigation of systems at increasingly smaller scales is hindered by optical diffraction, which limits the maximum optical forces that can be achieved in an optical tweezer for a given input power [9]. This is particularly salient for biological systems, wherein high input optical power can lead to specimen damage [10], [11]. Recently, plasmonic optical tweezers have emerged as a promising avenue to circumvent this issue. Also known as plasmonic “nanotweezers”, this architecture employs metallic nanoantennas to concentrate and enhance incident optical fields in deep-subwavelength gaps [12]–[17]. This yields large near-field intensity gradients that greatly amplify optical forces for a given input power [12], [13], enabling strong optical trapping with input-power densities 2–3 orders of magnitude lower than the biological damage threshold [18].

Following this reasoning, there have been several studies employing plasmonic nanostructures to trap biological objects. For instance, Righini *et al*. showed that living *Escherichia coli* bacteria can be stably trapped in a plasmonic nanotweezer comprised of dipole nanoantennas for more than two hours without visible damage [10]. Similarly, studies by Huang *et al*. and Miao and Lin demonstrated plasmonic trapping of yeast cells using a microfluidic platform containing Au nanodisks [19] and a spherical Au nanoparticle array [20], respectively. Despite these initial experimental demonstrations, no studies exist to date that systematically address the impact of biologically relevant buffers on the trapping capabilities of either standard or plasmonic-based tweezers. Biological buffers (media) are critical to *in vitro* studies in order to mimic the biological environment outside of a host organism. As a result, such buffers are often designed to operate, e.g., at specific atmospheric conditions (%CO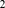), physiologically relevant temperature, and pH, and thus should not be ignored in calibration of optical trapping platforms used for biophysical assays.

In this paper, we investigate the effects of five widely used, biologically relevant media (3 cell growth media and 2 buffers) on the trapping performance of both plasmonic nanotweezers that are based on Au bowtie nanoantenna arrays (BNAs) and conventional high-numerical aperture (NA) optical tweezers. We perform high-precision, temperature-dependent rheological measurements on the media to determine their viscosity and assess trapping performance by measuring the optical trapping stiffness on 1.5-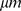 diameter polystyrene spheres in the various media. The effects of the medium pH and nanostructure geometry on trap stiffness are investigated. Our results show that the main contributor to the variation in performance of plasmonic nanotweezers in the biological media is the viscosity. Moreover, we show that in the biological media, plasmonic trapping strength is up to 4x that of conventional optical tweezers and is not mitigated compared to the water-only environment commonly used for trapping experiments. The cell growth solutions used in this study are utilized in cancer research, cardiovascular research, and common molecular biology assays. These findings have important implications for making plasmonic optical trapping more accessible to biological studies.

## Experimental Methods

### Biological Buffer Preparation

The breast cancer cell media (BC) is comprised of high-glucose Dulbecco's Modified Eagle Medium (DMEM) containing 10% fetal bovine serum (Invitrogen, Carlsbad, CA) and 1% Penicillin-Streptomycin (Invitrogen, Carlsbad, CA). The DMEM contains sodium pyruvate as an energy source and it contains sodium bicarbonate and sodium phosphate for buffering; such buffering is necessary for cellular growth in a 5% CO2 environment (incubator). The addition of 1% antibiotics prevents bacterial contamination and the serum contains biomolecules necessary for cell growth and cellular interactions, including: growth factors, enzymes, proteins, fatty acids and lipids, amino acids and carbohydrates [21]. This media is commonly utilized for the growth of human and mouse tumor cells, fibroblasts, macrophages, and other cell types. One of the co-authors (Imoukhuede) has recently employed this media in the growth of human breast cancer cell line MDA-MB-231 [22].

The endothelial growth medium (EGM2) is optimized for growth of human macrovascular endothelial cells in culture and is supplemented by the EGM-2 SingleQuot Kit, which contains FBS, growth factors and other ingredients for accelerated growth of healthy endothelial cells. This media is commonly used in cardiovascular research, including studies of angiogenesis. We have recently employed this media in the growth of human umbilical vein endothelial cells (HUVEC) [22]–[24]. The Lebovitz media (L15) contains glucose, free base amino acids and is buffered at pH 7.8 by salts. It is designed to be used with cells in a non-CO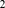 atmospheric conditions (outside an incubator).

In addition to these media, we also use two buffers in this study: Phosphate buffered saline (PBS) and Flow Cytometry Stain Buffer (Stain). Phosphate buffered saline (1x PBS, Fisher Scientific 10x power concentrate) is an aqueous solution consisting of Sodium Chloride (81%), Sodium Phosphate Dibasic (14%), and trace amounts of Potassium Phosphate Monobasic and Potassium Chloride. The ion concentrations and osmolarity of PBS are based on those found in the human body and the phosphate helps to buffer cell pH at 7.4 outside of an incubator. The Stain buffer is utilized for immunofluorescent staining of suspended cells and is a PBS-based solution with 2% bovine serum albumin (BSA), to reduce non-specific antibody bonding, and 0.09% of the preservative sodium azide. These buffers have fewer ingredients than the growth media and are widely used in flow cytometry applications. Each solution is prepared with two different pH values, 7.4 and 8.0, and the pH of the individual solutions is measured with a FiveEasy FE20 pH meter (Mettier-Toledo AG). A digital photograph of the media used in this study is shown in the supporting information (Fig. S1 in File S1).

### Viscosity Measurements

**Figure 1:**
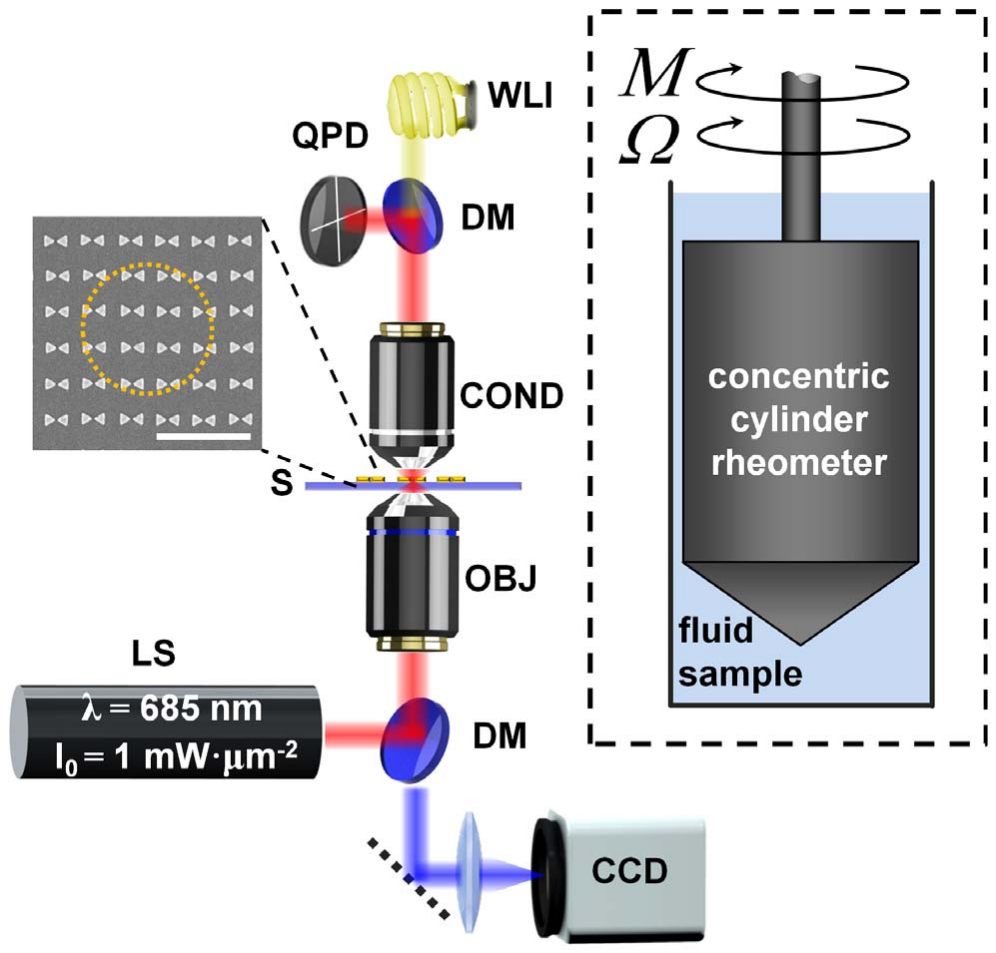
Schematic of the experimental setup. The experimental setup consists of a laser source (LS) coupled into the sample (S) by the microscope objective (OBJ) and dichroic mirror (DM). The sample inset shows a SEM image of the 425-nm array BNAs and the dotted-yellow line depicts the approximate focal spot diameter; scale bar is 1 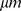. The condenser lens (COND) collects forward-scattered light from the trapped particle and the quadrant photodiode (QPD) detects Brownian fluctuations about the trap center. White-light illumination (WLI) provides visualization of particles on the CCD camera. The inset depicts the rotational rheometer geometry (not to scale) for the viscosity measurements. The measured torque *M* is due primarily to the simple shear flow in the thin gap between the inner rotor and outer stator. The shear viscosity 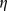 is calculated from the measured torque and angular velocity 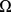.

Viscosity measurements of the biological media are performed using a rotational rheometer (Discovery Series Hybrid Rheometer (DHR), model HR-3, TA Instruments). The geometry is a single-gap, concentric cylinder (DIN standard) with conical bottom on the inner rotor. A schematic diagram is shown in the Fig. 1 inset. This geometry has shown highly reproducible results for shear-rate dependent measurements of low viscosity liquids, specifically because it minimizes surface tension torque effects that can appear inaccurately as shear-thinning [25]. The geometry has outer stator radius 30.35 mm, inner rotor radius 27.98 mm, and inner rotor working length 42.2 mm. A sample volume of 22.4 mL is used. Each sample is tested at temperatures of 20, 25, and 30 
C with Peltier temperature control at the outer surface. After loading, samples are held at the experimental temperature for 5 minutes prior to testing. Shear-rate sweeps are performed from 1 to 100 s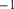 at T = 25 
C to determine the rate-dependent behavior of the biological media. Reported viscosity values are taken at 10 s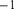 for the Newtonian samples and repeated in triplicate with separate sample loading to obtain precision error 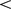 1%. For the measurably non-Newtonian Stain buffer at 8.0 pH, the reported viscosity is taken as the average from 2 to 50 s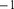 with no repeated measurements.

### Optical Trapping

The experimental optical trapping setup is built on an inverted microscope (Olympus IX-81) equipped with a 0.9-NA condenser (Olympus MPlanFL N 100x) that both provides white-light illumination for imaging trapped particles and collects the forward-scattered light from the trapping volume for trap stiffness measurements. The custom-built laser source is derived from a 685-nm wavelength laser diode that is spatially filtered and expanded to overfill the back-aperture of the microscope objective lens. For plasmonic optical trapping, a 0.6-NA objective (Olympus LUCPlanFLN 40x) is used to focus the incident beam onto the bowtie nanoantenna arrays (BNAs), which are fabricated onto a glass substrate with a 25-nm thick Indium-Tin-Oxide coating. The individual bowties comprising the BNAs are placed with two array spacings: 425 and 475 nm, which correspond to the center-to-center spacing between bowties along both *x* and *y* directions. Fabrication details can be found elsewhere [12]. The trapping chamber is formed using a 13-mm diameter gasket (Invitrogen) sandwiched between the BNA substrate and a rectangular #1 coverslip (Corning). The incident polarization is set parallel to the bowtie long axis in order to generate strong field concentration in the 20-nm gap. The chosen illumination wavelength is blue-detuned from the peak plasmon resonance of the BNAs, which produces strong optical forces without excessive plasmonic-absorption generated heating [15], [26].

Conventional optical trapping is performed using a 1.4-NA, oil-immersion objective (Olympus UPlanSApo 100x). The trapping chamber for conventional tweezers is formed by replacing the BNA substrate with a standard #1-1/2 coverslip (Corning). In all cases, the input power is adjusted to achieve a focal power density 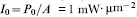, where the focal-spot area is given by 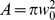, with focal-spot radius 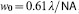, 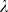 is the free-space, input wavelength, and 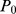 is the optical power measured at the focal plane. This process compensates for losses in the optical system. In the 0.6-NA case, 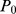 is directly assessed by placing an optical power detector near the focal plane, whereas for the 1.4-NA case, 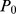 is assessed by re-collimating the focused laser with an identical objective and placing the power detector in the back-focal-plane of the objective. A schematic diagram of the experimental setup is shown in Fig. 1.

Optical trapping experiments are performed on 1.5-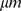 diameter polystyrene particles (Thermo Scientific), and the trap stiffness is assessed via the power spectrum method [27]. Here, a quadrant photodiode (QPD, Thorlabs PDQ80A) placed in the back-focal-plane of the condenser measures the position fluctuations of the trapped particle. The power spectrum of these Brownian fluctuations about the trap center is given by the Lorentzian [27]

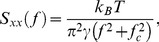
(1)where 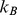 is Boltzmann's constant, *T* is the local temperature near the particle, 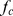 is the corner frequency, and 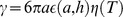 is Stokes' drag coefficient with particle radius *a* and temperature-dependent viscosity 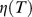 of the local fluid medium [27]. In order to account for the particle proximity to the substrate, we use the lubrication value of Faxen's correction 
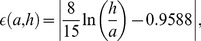
(2)where 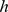 is the distance between the particle and the substrate [15], [28], [29]. In practice, it is difficult to determine 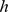 for a plasmonic trap, however, given the evanescent nature of plasmonic near-fields, particles must be within 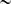
10–30 nm of the nanoantennas to experience enhanced optical forces [15], [30]. Thus, we use 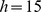 nm as an average value which gives 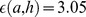; this value is assumed for both plasmonic and conventional trapping experiments. In the latter case, the particle height is set to within 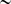 15 nm using a precision closed-loop microscope stage. Here, the axial position of the stage is moved with 10-nm precision until trapped particles are observed to contact the surface of the coverslip. Then, the stage is moved a single step away from the particle. The trap stiffness 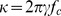 is then determined from the corner frequency obtained by fitting experimental power spectra to Eq. 1 via the Levenberg-Marquardt algorithm [27]. Position fluctuation signals are captured for 60 seconds using custom-written Labview software, and each corner frequency measurement represents the average of 15 independent measurements on the same particle, when possible.

## Results

**Figure 2:**
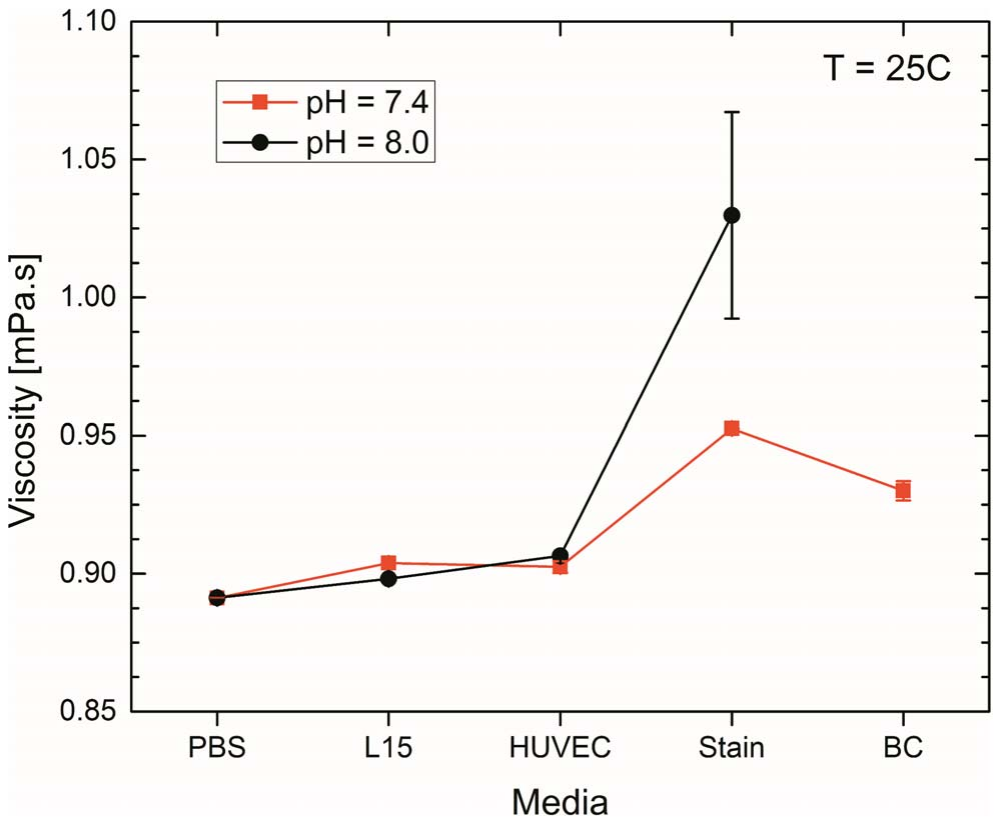
Viscosity measurement results. Experimentally measured viscosity data at 25

As a first step toward assessing the trap stiffness, we measure the steady-shear viscosities for the various media and the results are given in Fig. 2. Here, viscosity data are reported for T = 25 °C and a characteristic shear rate of 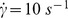; full temperature-dependent data are available in the supporting information (Fig. S2 in File S1). From the shear-rate dependent measurements, we find that all media (excluding Stain) exhibit Newtonian behavior for characteristic shear-rates 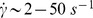. As a result, calculation of the trap stiffness utilizing the Stokes' drag coefficient 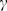 is justified [27], [29]. In contrast, the Stain media at pH  = 8.0 showed measurable shear-thinning behavior (Fig. S3 in File S1). This buffer includes bovine serum albumin protein, which may be stretched and oriented by shear flow and cause non-constant shear viscosity. We observed an approximate plateau viscosity (within 6%) over the range of 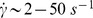 for this particular case, and therefore calculated viscosity from the new average within this range. For all the fluids tested, the pH has little effect on the viscosity, with the only appreciable deviation occurring for L15 which shows a 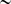 1% larger viscosity for pH  = 7.4. We note that the BC media is unstable and phase separates at 8.0 pH, and therefore no data is reported for this particular case.

C for the various media at 7.4 and 8.0 pH (red and black curves, respectively). Note the BC media is unstable at 8.0 pH and is therefore not included.

**Figure 3:**
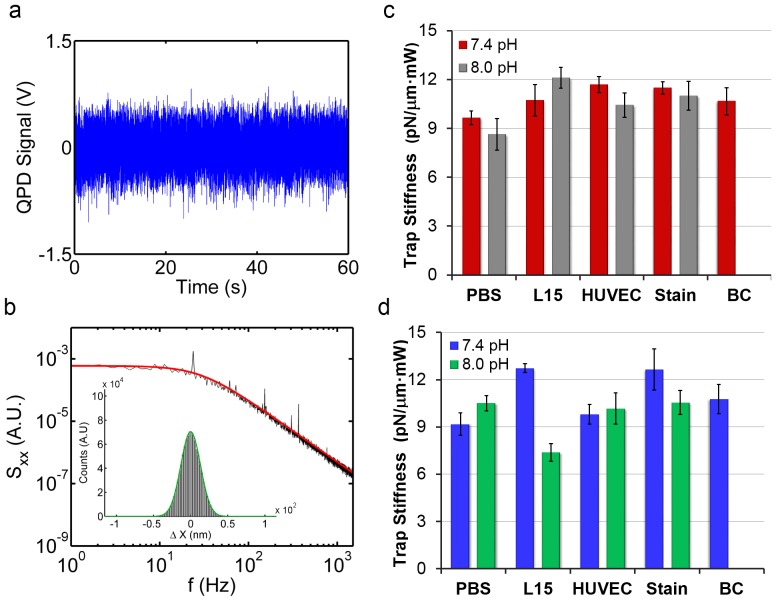
Plasmonic nanotweezer stiffness. Typical trap stiffness results showing (a) a time trace of the output from the quadrant photodiode and (b) the calculated power spectrum overlaid with a Lorentzian fit (red line). The inset shows a particle displacement histogram fit with a Gaussian curve. Measured trap stiffness for the biological media using plasmonic nanotweezers with (c) 425 and (d) 475-nm spaced BNAs. Error bars represent the standard error in stiffness measurements over 15 individual trials per data point.

The measured trap stiffness using the plasmonic optical tweezers for all parameters considered in this study are shown in Fig. 3. Figures 3a and 3b show typical trap stiffness results comprising the time trace of the QPD voltage signal and the calculated power spectrum with a Lorentzian fit, respectively. The inset in Fig. 3b shows a particle displacement histogram overlaid with a Gaussian fit; the close-fit of the histogram with the Gaussian indicates that the trapped particle experiences an approximate harmonic trapping potential, thereby validating the applicability of the trap stiffness model for plasmonic nanotweezers [15], [18]. Representative power-spectral particle displacement data for all medias are available in the supporting information (Fig. S4 in File S1). Figures 3c and 3d show the stiffness of the plasmonic optical traps using 425- and 475-nm spaced BNAs, respectively. It can be seen that for all cases, the plasmonic trapping stiffness varies between 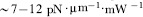, which is comparable to previously reported values in aqueous media [15], [18]. In calculating the stiffness, we use viscosity data taken at 25 °C (Fig. 2) due to heating effects by the plasmonic nanoantennas, which for the given input intensity results in an approximately 2–5°C temperature rise of the illuminated bowties [15], [26]. This indicates that the trapping performance of plasmonic nanotweezers is not significantly reduced in biologically relevant media. In most cases, there is no significant difference in the stiffness for the two pH values for a given media and the overall trend in trap stiffness follows that of the media viscosity reasonably well. This suggests that the most prominent cause for variation in trapping strength is the 5–10% variation in viscosity for the different media. Furthermore, the fact that 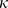 does not change as a function of pH implies that free ions in solution do not significantly alter the optical forces generated by the nanoantennas.

The minimal difference in stiffness between the two array spacings (for most cases) can be understood by comparing the relative near-field intensity enhancement, 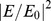 where *E* (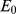) is the magnitude of the electric field generated by the nanoantennas (magnitude of the input electric field), and the absorption cross section data (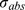) computed via Finite-Difference Time-Domain calculations [18]. Here, the intensity enhancement and absorption cross section serve as proxies for the maximum optical force and local heating, respectively. Comparing these values, we see that 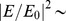 310 (200) for the 425 (475) array, whereas 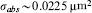 (0.015 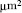) for the 425 (475) array. Thus, the 43% larger intensity enhancement, viz. optical force, for the 425 array is offset by a 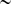
40% larger absorption cross section, which translates into higher local heating and thus enhanced Brownian perturbation to the trapped particle, i.e., lower trap stiffness. This effect has been previously observed in similar systems based on an aqueous solution [15], [18], which further indicates that general performance of the plasmonic system is retained when using biological media.

**Figure 4:**
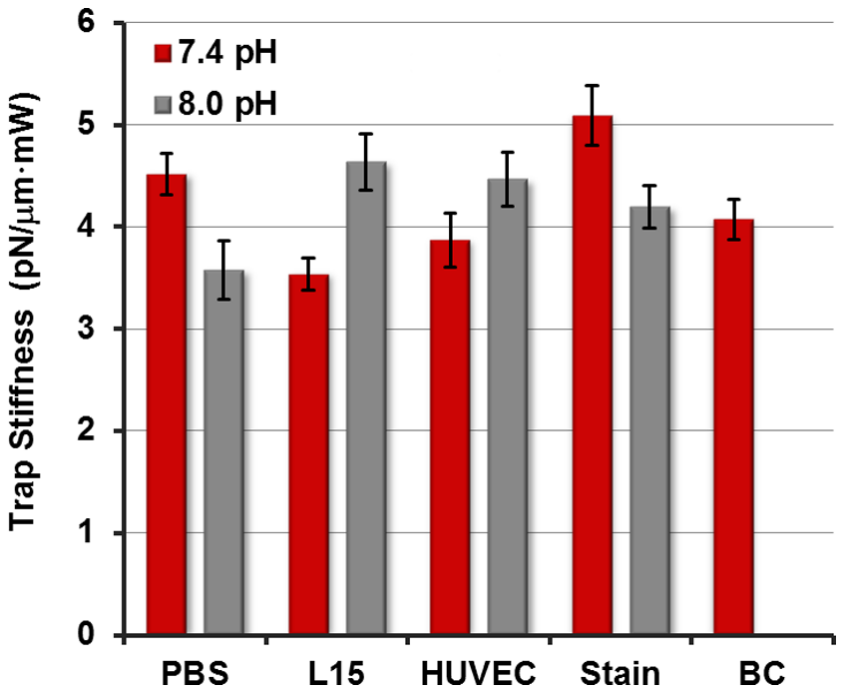
Conventional optical tweezer stiffness. Measured trap stiffness for the various media using a conventional optical tweezer. Error bars represent standard error in 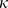 determination.

It is useful to compare the trap stiffness of the plasmonic nanotweezers with a conventional optical trap. Figure 4 depicts 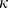 for a conventional optical trap based on a 1.4-NA objective. The overall lower stiffness obtained using conventional tweezers is clear, with 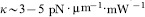. Interestingly, conventional tweezers display a stronger variation in trap strength as the pH is varied in contrast to the plasmonic case. A potential reason for this may be that the overall lower error in conventional stiffness measurements, which itself is due to reduced heating in this case, exposes more clearly the differences in optical force for the different pH values. Notwithstanding these differences, the benefit of using plasmonic nanotweezers compared to conventional tweezers in biological media is clear: the former produces larger trapping forces with lower input powers, thereby reducing potential phototoxic effects. Furthermore, these results suggest that the apparent higher sensitivity of standard optical tweezers to specific buffers is an important design criterion when choosing a platform for optical trapping-based biological studies.

## Discussion

Human physiological systems, along with almost all living things, are generally alkaline, water-based systems heavily reliant on acid-base equilibrium [31]. For this study, we choose the biologically relevant pH values 7.4 (typical) and 8.0 (maximal) due to the fact that optimal growth of mammalian cells is obtained at pH 7.2–7.4, human blood pH is regulated within the narrow range of 7.35 to 7.45, and mammalian cells are supported in the range of pH 6.6–7.8 [31], [32]. The presence of bovine serum albumin in the Stain, HUVEC, and BC trapping media has the potential to alter the dynamics of the trapped particle, given that BSA readily adsorbs onto many different surfaces due to the ease with which its structure changes [33]. Evidence for such an adsorption event would manifest as a variation of the corner frequency of the trap. However, examining the raw corner frequency data (Fig. S5 in File S1) reveals no correlation between the amount of experimental variation in the corner frequency and the percentage of BSA in the Stain, HUVEC, and BC media: 2%, 5%, and 10% weight by volume, respectively. As such, BSA adsorption likely does not significantly contribute to the measured trap stiffness.

For most cases, applying the measured viscosity data to the raw corner frequency data (see supporting information) results in trap stiffness values that follow the trend in media viscosity. However, the L15 media on the 475-nm array produce an anomalously high (low) trap stiffness for the 7.4 (8.0) pH samples. Similarly, the stain media do not produce significantly larger stiffness than the other media for both plasmonic and conventional optical traps, despite having the largest overall viscosities. Possible causes of these deviations include variations in material parameters such as the media refractive index, which alters the plasmon resonance and modifies the optical forces, or the thermal conductivity of the media, which changes the heat dissipation in the system. We note that determination of the trap stiffness from raw corner frequency data is strongly dependent on the value of 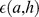, however, we apply the same value to both plasmonic and conventional trapping experiments. Moreover, variation between trapping systems is minimized by precisely controlling the axial position of particles in the conventional case. Given that 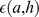 applies to the drag coefficient, it does not alter the corner frequency of the trap [34], and thus the 3–4x higher corner frequencies measured in the plasmonic case implies that the stiffness is indeed higher for plasmonic traps.

## Conclusion

We have shown that the trapping performance of plasmonic nanotweezers is largely unaltered when using biologically relevant media, producing trap stiffness values comparable to nanotweezers in a water-only environment and 3–4x higher than a conventional, high-NA optical tweezer. Our study confirms the Newtonian nature of several media commonly used in biological research via high-precision rheological measurements. In doing so, we validate the applicability of standard optical force-determination schemes (e.g., stiffness or drag-force efficiency) in biological media for both conventional and plasmonic optical tweezers. Variations in trap stiffness correspond reasonably well with trends in measured viscosity data, indicating that viscosity is the main factor contributing to the trap stiffness measured in a given medium.

## Supporting Information

File S1 **Supporting figures.** Figure S1, Digital photograph of the buffer media. Figure S2, Temperature-dependent viscosities. Figure S3, Shear-rate dependence of Stain medium. Figure S4, Representative power-spectral data. Figure S5, Raw corner frequency data.
(DOCX)
