# Supplementary material for: Plasmonic Optical Trapping in Biologically Relevant Media
Source: PLoS One. 2014 Apr 7;9(4):e93929. doi: 10.1371/journal.pone.0093929 (PMC3977964; doi:10.1371/journal.pone.0093929)
Supplement: File S1 — Supporting figures. Figure S1, Digital photograph of the buffer media. Figure S2, Temperature-dependent viscosities. Figure S3, Shear-rate dependence of Stain medium. Figure S4, Representative power-spectral data. Figure S5, Raw corner frequency data. (DOCX) [file pone.0093929.s001.docx]

**Plasmonic Optical Trapping in Biologically Relevant Media**

Brian J. Roxworthy^1^, Michael T. Johnston^2^, Felipe T. Lee-Montiel^3^, Randy H. Ewoldt^2^, Princess. I. Imoukhuede^3^, and Kimani C. Toussaint, Jr.^2,*^

^1^ Department of Electrical and Computer Engineering, University of Illinois at Urbana-Champaign, Urbana, IL, United States of America

^2^ Department of Mechanical Science and Engineering, University of Illinois at Urbana-Champaign, Urbana, IL, United States of America

^3^ Department of Bioengineering, University of Illinois at Urbana-Champaign, Urbana, IL, United States of America

* E-mail: [ktoussai@illinois.edu](mailto:ktoussai@illinois.edu)

**Supporting Information**

Figure S1 shows a digital photograph of the different buffer media used in this study.


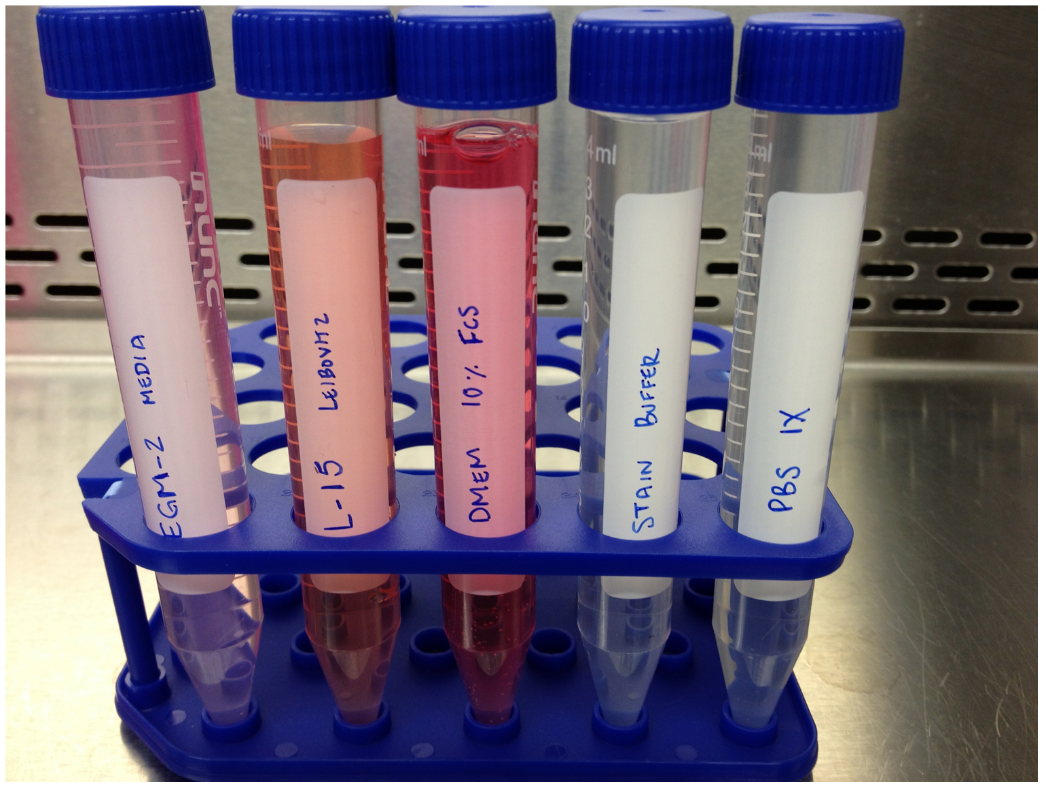


**Figure S1. Biological buffer media.** The media are (from left to right): EGM (HUVEC), L15, DMEM (BC), Stain, and PBS.

Figure S2 shows the temperature-dependent steady shear viscosities measured for the four Newtonian fluids used in this study (all fluids are Newtonian except the Stain buffer). Measurements are taken at a shear rate of 10 s^-1^ in triplicate with separate sample loading. The largest reported precision error is 4.7%.


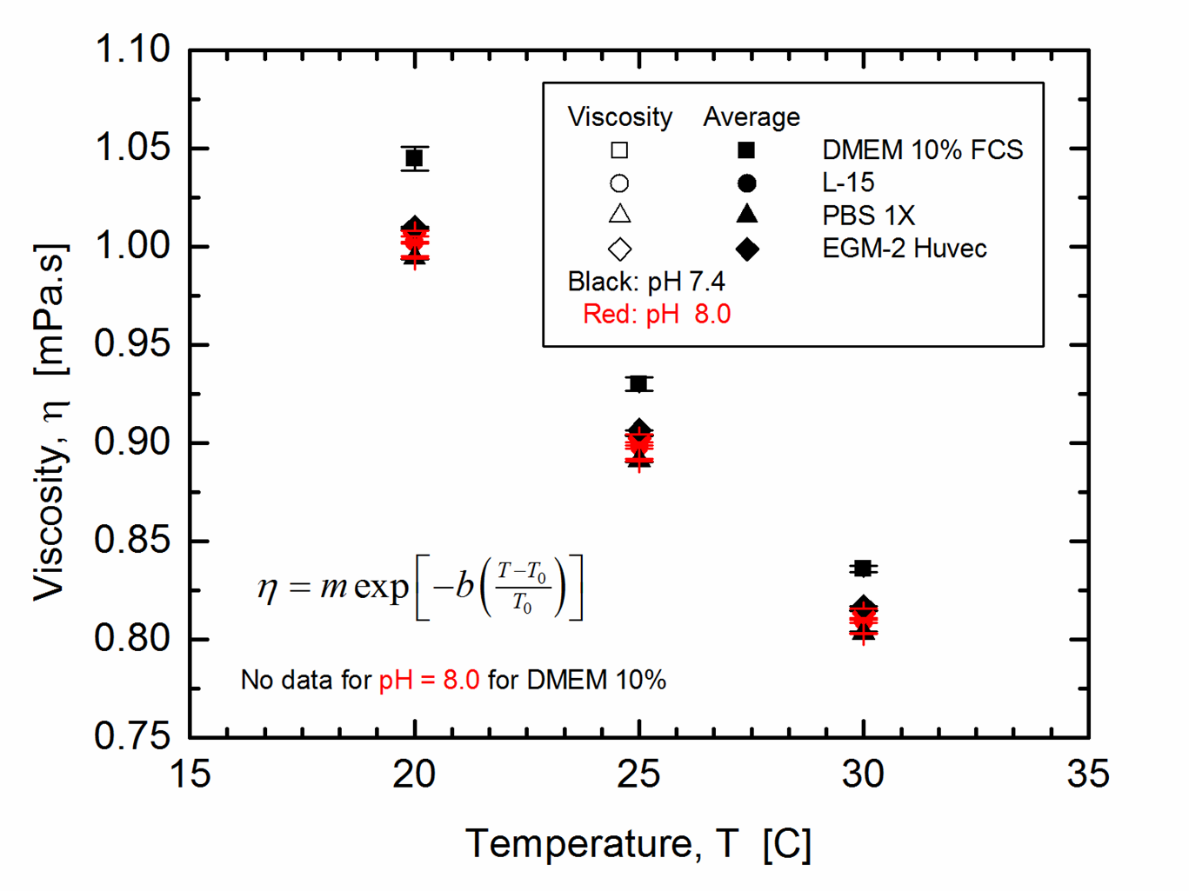


**Figure S2. Temperature-dependent viscosities.** Plot of average measured viscosity for all media considered in this study; data are taken at a shear-rate of 10 s^-1^. Three measurements of each buffer are taken at each temperature with error bars showing one standard deviation. The largest standard deviation is <1%.

Figure S3 shows the shear-rate dependent measurements of the Stain buffer with 8.0 pH. Measurements are taken from 0.05 to 50 s^-1^ with no repeated measurements. From approximately 2 to 50 s^-1^ a Newtonian plateau appears (for all test temperatures).


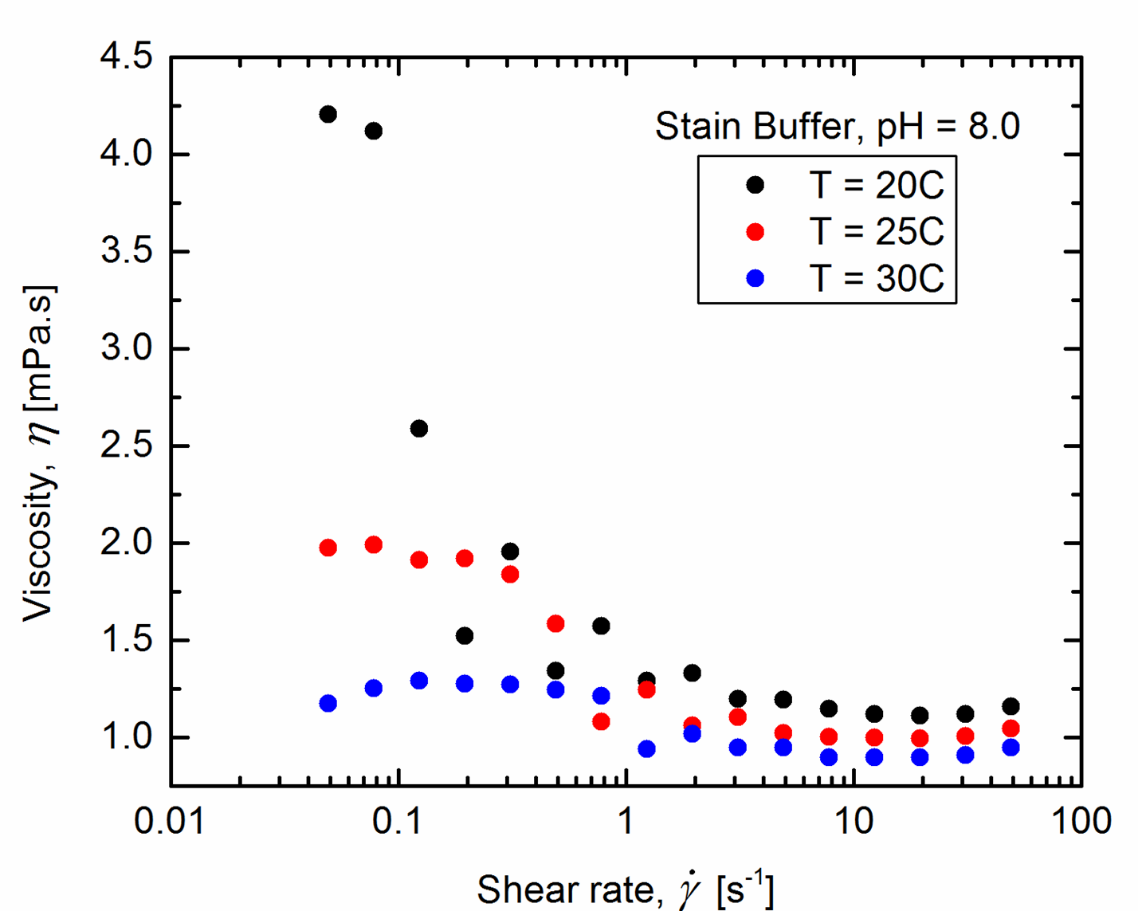


**Figure S3. Shear-rate dependence of Stain medium.** Plot of the shear-rate dependent viscosity measured for the 8.0 pH Stain buffer.

Figure S4 shows example power-spectral data (black curves) with Lorentzian fits overlaid (red curves). High-frequency peaks corresponding to electronic noise do not affect the fit. The insets show representative particle-displacement histograms fit with a Gaussian curve, included as the green line. The close fit for both curves indicates the validity of the trap stiffness measurements.


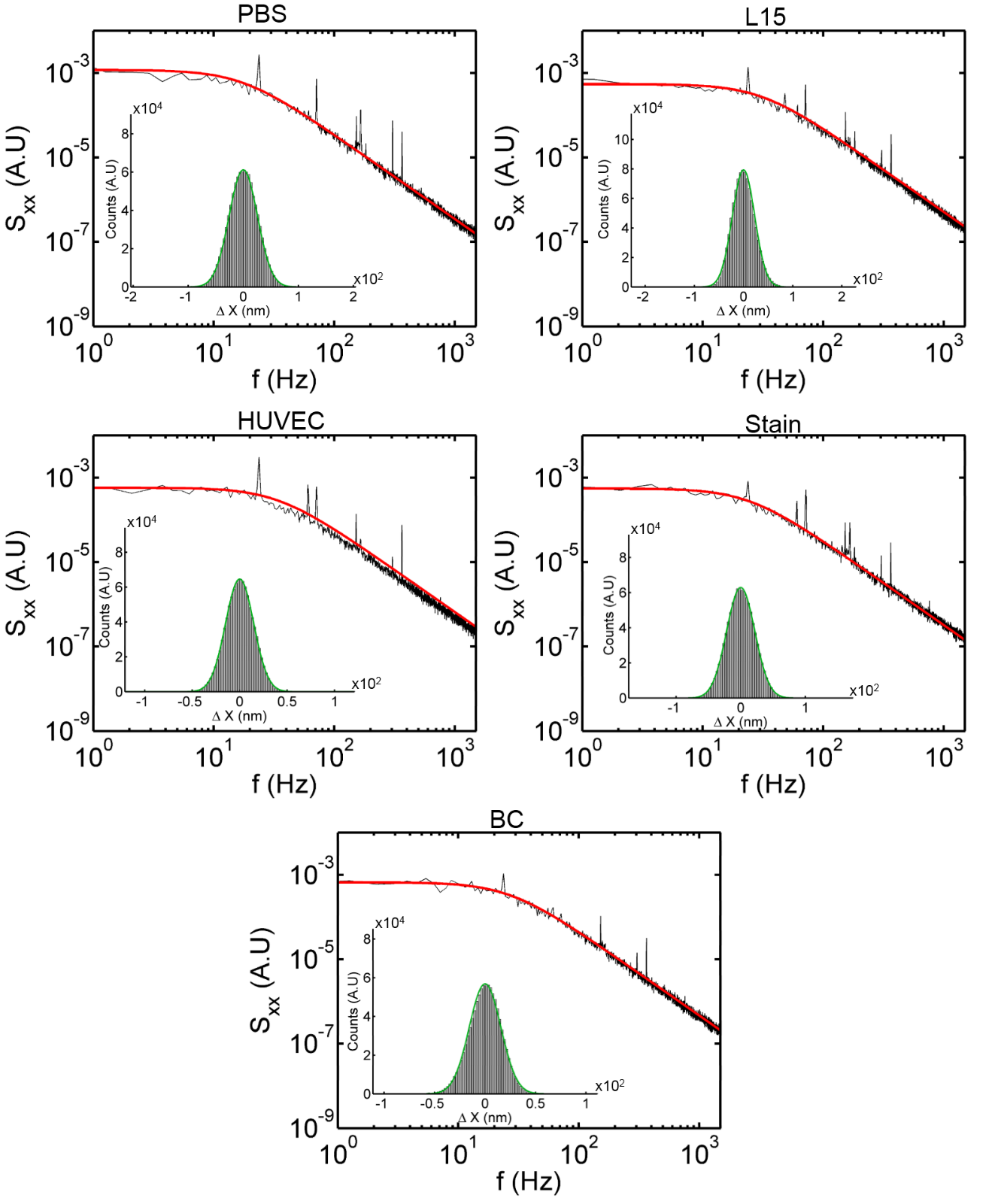


**Figure S4. Representative power-spectral data.** Plots of the experimentally measured particle-displacement power spectra (black lines) overlaid with Lorentzian fits (red lines). The insets show representative particle-displacement histograms with Gaussian fits.

Figure S5 shows the raw corner frequency data calculated from the quadrant-photodiode voltage-time trace.


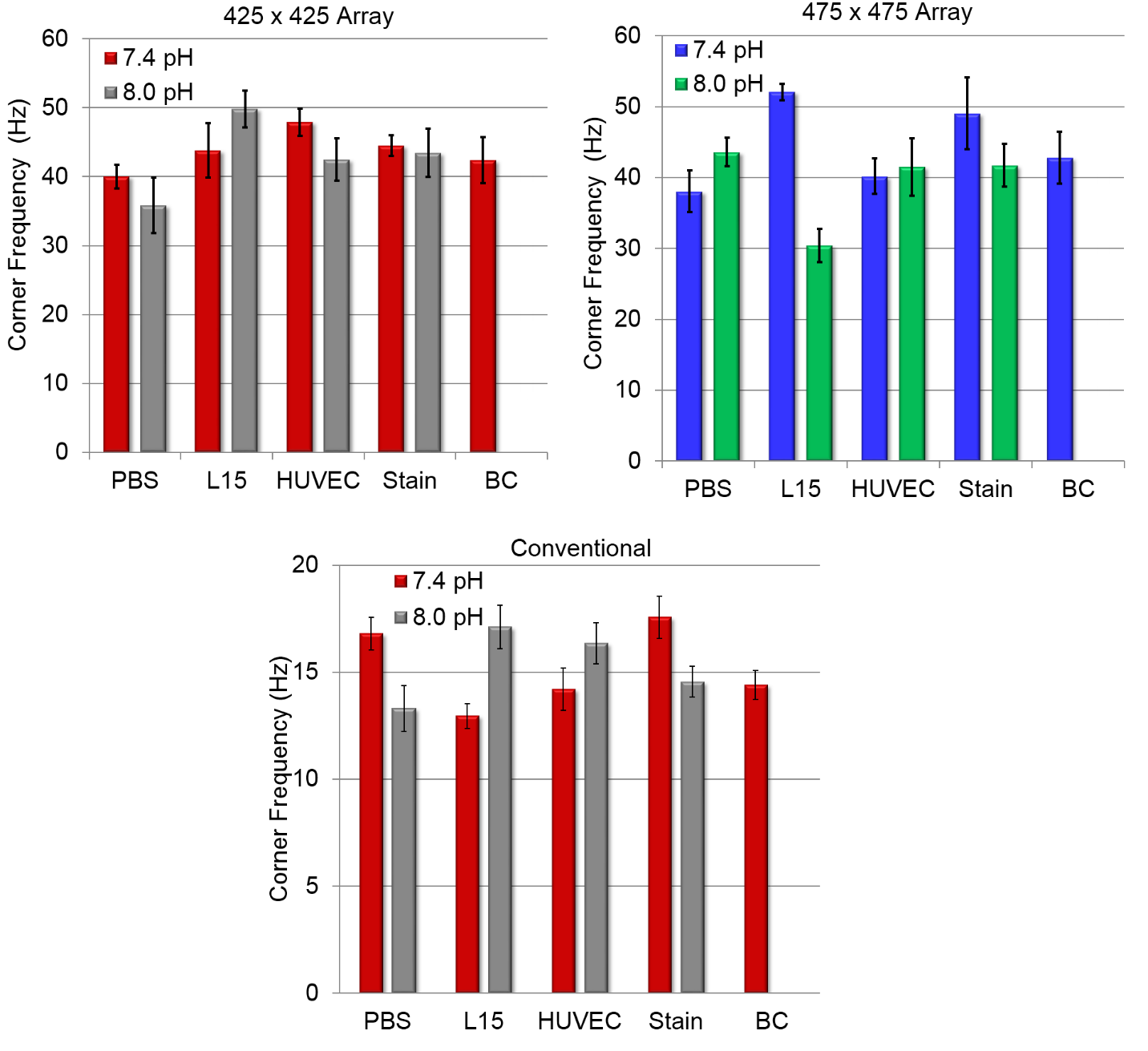


**Figure S5. Raw corner frequencies.** Plot of the raw corner frequency data for all media and pH values.
